# Time limit and V̇O_2_ kinetics at maximal aerobic velocity: Continuous vs. intermittent swimming trials

**DOI:** 10.3389/fphys.2022.982874

**Published:** 2022-09-30

**Authors:** Tiago A. F. Almeida, Danilo A. Massini, Osvaldo T. Silva Júnior, Rubens Venditti Júnior, Mário A. C. Espada, Anderson G. Macedo, Joana F. Reis, Francisco B. Alves, Dalton M. Pessôa Filho

**Affiliations:** ^1^ Department of Physical Education, São Paulo State University—UNESP, São Paulo, Brazil; ^2^ CIPER, Faculdade de Motricidade Humana, Universidade de Lisboa, Lisboa, Portugal; ^3^ Postgraduate Program in Human Development and Technologies, São Paulo State University—UNESP, Rio Claro, São Paulo, Brazil; ^4^ Instituto Politécnico de Setúbal (CIEF—ESE/IPS, CDP2T, ESTSetúbal/IPS), Setúbal, Portugal; ^5^ Life Quality Research Centre (CIEQV-Leiria), Santarém, Portugal; ^6^ Laboratory of Physiology and Biochemistry of Exercise, Faculdade de Motricidade Humana, Universidade de Lisboa, Lisboa, Portugal

**Keywords:** maximal aerobic velocity, interval training, VO_2_ response, time-limit, swimming

## Abstract

The time sustained during exercise with oxygen uptake (V̇O_2_) reaching maximal rates (V̇O_2peak_) or near peak responses (i.e., above second ventilatory threshold [t@VT_2_) or 90% V̇O_2peak_ (t@90%V̇O_2peak_)] is recognized as the training pace required to enhance aerobic power and exercise tolerance in the severe domain (time-limit, t_Lim_). This study compared physiological and performance indexes during continuous and intermittent trials at maximal aerobic velocity (MAV) to analyze each exercise schedule, supporting their roles in conditioning planning. Twenty-two well-trained swimmers completed a discontinuous incremental step-test for V̇O_2peak_, VT_2_, and MAV assessments. Two other tests were performed in randomized order, to compare continuous (CT) vs. intermittent trials (IT_100_) at MAV until exhaustion, to determine peak oxygen uptake (Peak-V̇O_2_) and V̇O_2_ kinetics (V̇O_2_K). Distance and time variables were registered to determine the t_Lim_, t@VT_2_, and t@90%V̇O_2peak_ tests. Blood lactate concentration ([La^−^]) was analyzed, and rate of perceived exertion (RPE) was recorded. The tests were conducted using a breath-by-breath apparatus connected to a snorkel for pulmonary gas sampling, with pacing controlled by an underwater visual pacer. V̇O_2peak_ (55.2 ± 5.6 ml·kg·min^−1^) was only reached in CT (100.7 ± 3.1 %V̇O_2peak_). In addition, high V̇O_2_ values were reached at IT_100_ (96.4 ± 4.2 %V̇O_2peak_). V̇O_2peak_ was highly correlated with Peak-V̇O_2_ during CT (*r* = 0.95, *p* < 0.01) and IT_100_ (*r* = 0.91, *p* < 0.01). Compared with CT, the IT_100_ presented significantly higher values for t_Lim_ (1,013.6 ± 496.6 vs. 256.2 ± 60.3 s), distance (1,277.3 ± 638.1 vs. 315.9 ± 63.3 m), t@VT_2_ (448.1 ± 211.1 vs. 144.1 ± 78.8 s), and t@90%V̇O_2peak_ (321.9 ± 208.7 vs. 127.5 ± 77.1 s). V̇O_2_K time constants (IT_100_: 25.9 ± 9.4 vs. CT: 26.5 ± 7.5 s) were correlated between tests (*r* = 0.76, *p* < 0.01). Between CT and IT_100_, t_Lim_ were not related, and RPE (8.9 ± 0.9 vs. 9.4 ± 0.8) and [La^−^] (7.8 ± 2.7 vs. 7.8 ± 2.8 mmol·l^−1^) did not differ between tests. MAV is suitable for planning swimming intensities requiring V̇O_2peak_ rates, whatever the exercise schedule (continuous or intermittent). Therefore, the results suggest IT_100_ as a preferable training schedule rather than the CT for aerobic capacity training since IT_100_ presented a significantly higher t_Lim_, t@VT_2_, and t@90%V̇O_2peak_ (∼757, ∼304, and ∼194 s more, respectively), without differing regards to [La^−^] and RPE. The V̇O_2_K seemed not to influence t_Lim_ and times spent near V̇O_2peak_ in both workout modes.

## Introduction

The maximal aerobic velocity (MAV), which corresponds to the minimal velocity at which the maximal oxygen consumption of an athlete occurs, is one of the most important variables of study in sports physiology since it combines exercise economy and maximal V̇O_2_ rates into a single factor, being well related with performance (Billat and Koralsztein, 1996; Demarie et al., 2000; Reis et al., 2012; Espada et al., 2015; Almeida et al., 2021). This velocity, associated with the 3,000 m running (Lacour et al., 1990; Demarie et al., 2000) or the 400 m swimming (Espada et al., 2015; Zacca et al., 2019) velocities, is usually used by coaches for training intensity prescriptions (Demarie et al., 2000; Fernandes and Vilas-Boas, 2012; Espada et al., 2015; Zacca et al., 2019,^2020^). Therefore, studying the time to exhaustion (t_Lim_) at MAV (t_Lim_-MAV) is extremely important, primarily to provide insightful information regarding the athletes’ capacity at this intensity, aiming for better planning of the training sets (Fernandes et al., 2008). Moreover, it is generally accepted that exercise intensities between 70% and 100% of V̇O_2_ maximal rates, as well as training sets sustained near V̇O_2_ maximal rates have been reported to improve the aerobic power (Billat and Koralsztein, 1996; Demarie et al., 2000; Millet et al., 2003; Almeida et al., 2021), and therefore also improve long term performance (Bentley et al., 2005; Libicz et al., 2005).

It is well recognized that how fast an athlete can reach each exercise’s energetic requirements will contribute to its oxidative response, reducing metabolites accumulation, and delaying the fatigue process (Jones and Poole, 2005). In this sense, faster primary V̇O_2_ responses have been associated with higher conditioning levels (Jones and Burnley, 2009; Reis et al., 2012; Espada et al., 2015), as well as related to the time spent near V̇O_2_ maximal values during interval training (IT) running sessions (Millet et al., 2003). However, only two studies analyzed continuous V̇O_2_ response in IT swimming sessions (Bentley et al., 2005; Almeida et al., 2021).

Previous studies which analyzed the exercise tolerance around MAV have shown an interesting inverse relationship between t_Lim_-MAV with the MAV and the velocity of the second ventilatory threshold (vVT_2_), which seems to suggest that high-level athletes could have a lower capacity to deal with this relative intensity (Billat et al., 1996; Billat and Koralsztein, 1996; Faina et al., 1997; Fernandes et al., 2008; Fernandes and Vilas-Boas, 2012). Also, the relationship between t_Lim_-MAV with the V̇O_2_ slow component and V̇O_2peak_ seems not to be a consensus in the literature regarding the positive relationship between higher V̇O_2_ slow component and V̇O_2peak_ with longer times to exhaustion (Billat and Koralsztein, 1996; Billat et al., 1998; Demarie et al., 2001; Fernandes et al., 2003, 2008; Fernandes and Vilas-Boas 2012). Furthermore, there is a lack of studies that can translate the t_Lim_-MAV characteristics to other real training situations such as interval training in swimming; but being one of the few, the study of Demarie et al. (2000) reported higher t_Lim_ and times spent near V̇O_2_ maximal values in IT compared to the continuous running trial.

The current study aimed to compare physiological responses during two different training modes—continuous (CT) vs. intermittent (IT_100_) swimming sets both performed until exhaustion (t_Lim_), in order to verify the differences regarding the t_Lim_ and times spent near V̇O_2peak_. We hypothesize that: 1) both time-limit tests will promote a high V̇O_2_ response near V̇O_2_ maximal values, and therefore recognize both conditions as suitable schedules for training to improve maximal cardiorespiratory conditioning; 2) the IT_100_ will present a higher t_Lim_ and a longer time spent near V̇O_2_ maximal values, which is an expectance when considering the recognized effect of IT mode of exercise on reducing metabolites accumulation (Zuniga et al., 2011; Rønnestad & Hansen, 2016; Almeida et al., 2021); and 3) faster V̇O_2_ responses will be related with longer times to exhaustion in the time-limit tests, since the assumption relating t_Lim_ to V̇O_2_ kinetics considers that fast V̇O_2_ response to target muscle O_2_ requirements would reduce O_2_ deficit and metabolite accumulation, and increase oxidative contribution (Bailey et al., 2009).

## Materials and methods

### Participants

Twenty-two well-trained swimmers (9 females and 13 males), were informed about the procedures and experimental risks and gave their written informed consent (and the respective legal guardians, when they were under 18 years old) in order to participate in this study. *A priori* sample N was determined with G*Power 3 from data including five participants (three males and two females) of time above VT_2_ (CT: 146.5 ± 120.3 vs. IT_100_: 268.6 ± 88.4 s), and specifying *α* = 0.05 and 1-β = 0.80 (Faul et al., 2007). The output N = 20 was further increased by 10% to consider possible withdrawal from the study, totalizing 22 participants.

The swimmers showed time performance within 20% of the world record, therefore the “highly trained/national level” matched the conditioning profile of the current sample of participants, as recommended in McKay et al. (2022). In addition, the current swimmers planning includes seven to eight training sessions which total ∼32 km per week in water, as well as dry land workouts. Also, the current swimmers had been regularly involved with competitive events for at least 3 years prior the study. All swimmers were fully familiarized with the equipment and the test procedures before the test sessions, being frequent participants in similar experimental studies that our research group has undertaken. This study was approved by the local University Ethical Committee (CEFMH: 39/2015) and conducted following the 1964 Declaration of Helsinki (Harriss et al., 2017). The descriptive characteristics of the swimmers are presented in Table 1.

**TABLE 1:** Mean ± SD of the descriptive characteristics of the swimmers.

Variables	Female	Male	Group
Age (years)	15.3 ± 1.2	16.5 ± 1.9	16.1 ± 1.7
Height (cm)	165.0 ± 6.5	178.6 ± 8.4	173.0 ± 10.2
Body Mass (kg)	58.4 ± 6.0	70.4 ± 10.3	65.5 ± 10.6
PB 200 (s)	122.2 ± 5.9	136.8 ± 5.7	65.5 ± 10.6
% to WR	∼19.6	∼21.2	-

### Experimental design

All swimmers performed three testing sessions, separated by at least 48 h: 1) a discontinuous incremental step-test; and 2) two time-limit sessions at the MAV intensity, a continuous test (CT) vs. an intermittent test (IT_100_). All subjects performed the same pre-test warm-up protocol, which followed the schedule suggested in Almeida et al. (2020), e.g., dry land stretching exercises for upper- and lower-limbs, and 800 m swimming at a comfortable and effortless pace, including whole-body, and only arms and legs swimming practices. The swimmers were instructed to avoid strenuous exercise in the preceding 24 h before each session, attend well hydrated and fed, and abstain from caffeine and alcohol in the preceding 24 h. In order to minimize the effect of circadian rhythms or differences in prior exercise, the same environmental conditions were applied to all tests, namely the time of day (±2 h), water temperature (∼28°C), and relative humidity (∼50%).

A telemetric portable breath-by-breath gas analyzer (K4b^2^, Cosmed, Italy), connected to the swimmer by a respiratory snorkel and valve system (new-AquaTrainer^®^, Cosmed, Italy), was used in all tests in order to measure the respiratory and gas exchange variables for cardiorespiratory analysis (Reis et al., 2010; Baldari et al., 2013). The K4b^2^ was calibrated before each test according to the manufacturer’s instructions. All tests were performed in front crawl swimming with in-water starts and open turns without underwater gliding.

The heart rate (HR) was telemetrically recorded during exercise with an HR monitor (Polar^®^, Finland) coupled to the snorkel and synchronized with the K4b^2^ system. For the blood lactate concentration [La^−^] analysis a biochemistry analyzer was used (YSI, 2300 STAT, Yellow Springs, United States), and capillary blood samples (25 μl) were collected from the earlobe before the start of each test, during the breaks of the discontinuous incremental step-test and at 1, 3, 5, and 7 min after all tests. The option for the earlobe site considered the assumption that the [La^−^] analysis did not differ between sample sites, particularly when movement involved both legs and arms, and is performed at high exercise intensity (Forsyth and Farrally, 2000). The rate of perceived exertion (RPE) was also recorded through the Borg’s CR-10 scale (Borg, 1990).

An underwater visual pacer (Pacer2Swim^®^, KulzerTEC, Portugal) was placed along the bottom of the pool for the swimming velocity control. This system is composed of 26 lights that subsequently light up, giving the swimmer an accurate notion of the correct velocity for each test. For time-limit tests, a tolerance of 2% of the overall time was given to the swimmers. Tests were finished when the swimmers exceeded the tolerance or when individual voluntary exhaustion was observed.

The sessions were performed in a 25 m swimming pool at the beginning of the preparatory period of the second macrocycle of the swimmers’ competitive season, after 2 weeks of training adaptation.

### Incremental step-test

This test was composed of six sets of 250 m, plus one set of 200 m at maximal intensity, with 30 s rest for [La^−^] collection (Espada et al., 2015; Almeida et al., 2020, 2021; Massini et al., 2021), in order to allow the determination of maximal oxygen uptake (V̇O_2peak_), VT₂, vVT₂, and MAV. The velocity of the first repetition was set at 50% of the swimmers’ 200 m trial velocity (performed 48 h before the beginning of the tests), and increments of 5%–10% were imposed in the remaining repetitions until swimmers’ voluntary exhaustion. V̇O_2peak_ was recorded as the highest 30 s average of the V̇O_2_, and MAV was considered the minimal velocity at which the V̇O_2peak_ values were reached (both reached in the last two repetitions).

### Time-limit sessions

**FIGURE 1:**
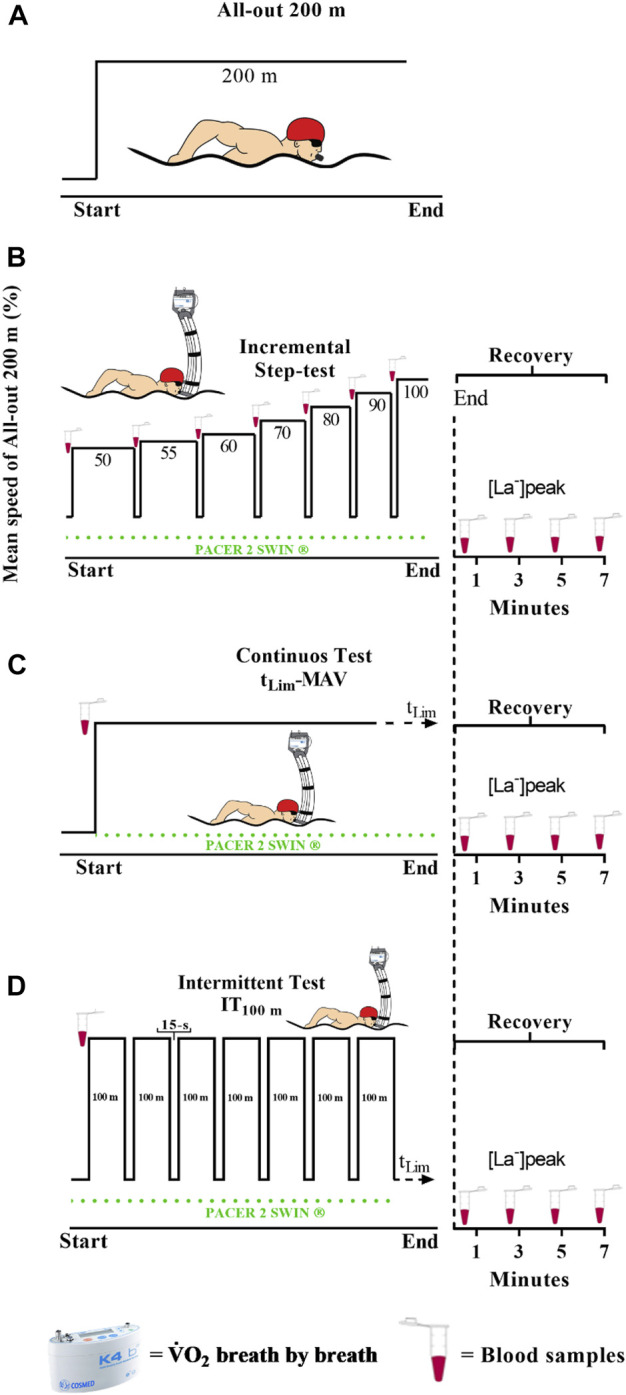
Overview of experimental design for 200 m performance **(A)**, discontinuous incremental step-test **(B)**, continuous test **(C)**, and intermittent test **(D)**.

In subsequent days, in a randomized order, the swimmers performed two time-limit sessions at MAV until exhaustion: 1) a constant load set (CT); 2) and an interval set composed of 100 m repetitions (IT_100_), with 15 s breaks with passive rest. In both sessions, the t_Lim_ and distance were recorded. The selected planning for the IT protocol was supported by the findings that short (i.e., 100 m) or long (i.e., 200 m) work intervals did not differ with regard to physiological and temporal responses at MAV condition in swimming, but the shortest is perceived as less difficult to perform and therefore suitable to ensure swimmer engagement at such an exhaustive training condition (Almeida et al., 2021). Apart from the option for the ideal IT distance, the work:rest ratio for IT_100_ followed the recommendations of Billat, (2001), which suggested 10–30 s of rest to training for high intensity aerobic short-intervals, considering that 1) rest should be long enough to ensure the restoration of the O_2_ reserve and phosphocreatine sources partially, but 2) short enough to avoid considerable reduction of V̇O_2_. The maximal V̇O_2_ response (Peak-V̇O_2_), oxygen deficit at the onset of exercise (O_2InitialDef_), maximal [La^−^], and the V̇O_2_K parameters were determined (we use the first bout in the IT_100_ session to compare with the CT). Additionally, the time spent at or above the VT₂ (t@VT₂) and 90% of the V̇O_2peak_ (t@90%V̇O_2peak_), and the corresponding percentage values for the total duration of the sessions, were registered (%t@VT₂ and %t@90%V̇O_2peak_, respectively). For the IT_100_, the mean Peak-V̇O_2_ (MPeak-V̇O_2_) as the average value of the Peak-V̇O_2_’s of each repetition was calculated. The swimmers were encouraged to give their maximal effort in the incremental test and perform the maximal distance in the time-limit tests. Figure 1 depicts the overall view of all testing protocols.

### Data analysis

Breath-by-breath V̇O_2_ data were first cleaned by the exclusion of values lying more than three standard deviations from the local mean for the exclusion of outliers caused by abrupt breaths or coughing. For maximal oxygen uptake determination, a 30 s moving average of data was used for the incremental and time-limit tests considering the highest value as the peak. For the t@VT₂, t@90%V̇O_2peak_, and respective percentage values for the total duration of the sessions, V̇O_2_ data was further interpolated into 1 s values, and all the above values were registered.

V̇O_2_K parameters [time delay (TD), time constant (τ), and amplitude (A)] of the time-limit tests were determined by using: 1) bi-exponential modelling for the CT, since after a primary rise of the V̇O_2_ values, a secondary rise (slow component) was observed (except for two swimmers); or 2) by monoexponential modelling for the IT_100_, since due to the short duration of the sets we did not observe the secondary rise of the V̇O_2_ values, in accordance with previous studies (Rodríguez et al., 2003; Sousa et al., 2013; Almeida et al., 2020, 2021). To remove the influence of the cardiodynamic phase on the subsequent V̇O_2_ response, we chose to remove the first 20 s of data from the analysis (Pessôa Filho et al., 2012; Reis et al., 2012; Espada et al., 2015; Almeida et al., 2020, 2021). We also calculated an individual “snorkel delay” (ISD) for each test, as described previously by Reis et al. (2012), adapted to the specific characteristic of the snorkel device used in this study.

V̇O_2_K parameters were calculated through an iterative procedure by minimizing the sum of the mean squares of the differences between the modelled and the measured V̇O_2_ values. Therefore, we modelled the V̇O_2_K according to the equation (Jones and Poole, 2005):

V̇O_2(t)_ = V̇O_2(b)_ + A_p_ • (1-e^-(t−TDp)/*τ*p^) + A_sc_ • (1-e^-(t−TDsc/*τ*sc)^)Where V̇O_2(t)_ represents the relative V̇O_2_ at a given time; V̇O_2base_ represents the V̇O_2_ at rest, which was calculated as the average of the first 30 s of the last minute before the start of the exercise (after 10 min of passive rest); TD, τ, and A represent the time delay, the time constant (time that is needed to complete 63% of the V̇O_2_ response), and the amplitude of the exponential response of the V̇O_2_, respectively for the primary (p) and the slow component (sc) phases.

### Statistical analysis

Firstly, normality and homogeneity of data were confirmed with Shapiro-Wilk and Levene tests; secondly, independent T-tests were applied to variables to check the differences between sexes. The differences between V̇O_2peak_ values observed during the discontinuous incremental step-test and the CT and IT_100_ tests were tested for statistical significance using one-way ANOVA with Sidak post-hoc analysis. The independent Student’s t-test analyzed the differences between sexes with regards to conditioning parameters, as well as being used to test for differences between the time-limit tests. The effect size for each Student’s *t*-test comparison was determined by Hedges’ g, which is considered: <0.19 (trivial), 0.20–0.49 (small), 0.50–0.79 (medium), 0.80–1.29 (large), and >1.30 (very large) (Rosenthal, 1996). The sample power was determined considering the security level at 95% (*α* = 0.05), and a minimal power at 80% (1 - *β* = 0.80) to satisfy the confidence of the differences between sexes and training trials, when observed. Lastly, Pearson’s linear correlation coefficient was used to establish the significant associations between physiological measures and swimmers’ performance in the time-limit tests. Statistical significance was accepted at *p* < 0.05. All statistical comparisons were performed with the Statistical Package for the Social Sciences (version 25.0; SPSS, Chicago, IL, United States), and power analysis was estimated with G*Power 3 software.

## Results

The physiological responses of the swimmers in the incremental test are depicted in Table 2. Except for the swimming velocities, as expected, no differences were found between sexes with regards to the conditioning parameters.

**TABLE 2:** Mean ± SD of the conditioning parameters assessed during incremental test, by sex and group.

	Sex			Power	
**Variables**	**Group**	**Female**	**Male**	**ρ**	**Hedges’ g**
V̇O_2peak_ (ml·kg^−1^·min^−1^)	55.2 ± 5.6	52.5 ± 4.2	57.0 ± 5.7	0.054	0.80 [large]
VT₂ (ml·kg^−1^·min^−1^)	48.4 ± 5.0	46.4 ± 4.3	49.9 ± 4.8	0.107	0.71 [medium]
%VT₂ (%V̇O_2peak_)	87.9 ± 3.2	88.3 ± 2.5	87.6 ± 3.5	0.603	0.20 [trivial]
vVT₂ (m·s^−1^)	1.19 ± 0.08	1.11 ± 0.04	1.24 ± 0.06*	<0.001	1.99 [very large]
MAV (m·s^−1^)	1.26 ± 0.09	1.20 ± 0.07	1.30 ± 0.07*	0.007	1.37 [very large]
Peak [La^−^] (mmol·l^−1^)	8.4 ± 3.3	7.9 ± 2.5	8.8 ± 3.6	0.178	0.27 [small]
Peak HR (b·min^−1^)	184.1 ± 9.4	188.7 ± 9.2	180.4 ± 7.8	0.059	0.95 [large]

V̇O_2peak_, maximal oxygen uptake; VT₂ and %VT₂, V̇O_2_ at the second ventilatory threshold and corresponding percentage value for V̇O_2peak_; vVT₂, velocity at VT₂; MAV, maximal aerobic velocity; Peak [La^−^], maximal blood lactate concentration; Peak HR, maximal HR; *, statistical differences for the female group (*p* < 0.05). The observed sample power for the differences between sexes with regards to vVT_2_ and MAV are 100 and 88%, respectively. For the other variables, The differences between sexes neither attained statistical significance or sufficient sample power (i.e., <80 %).

**FIGURE 2:**
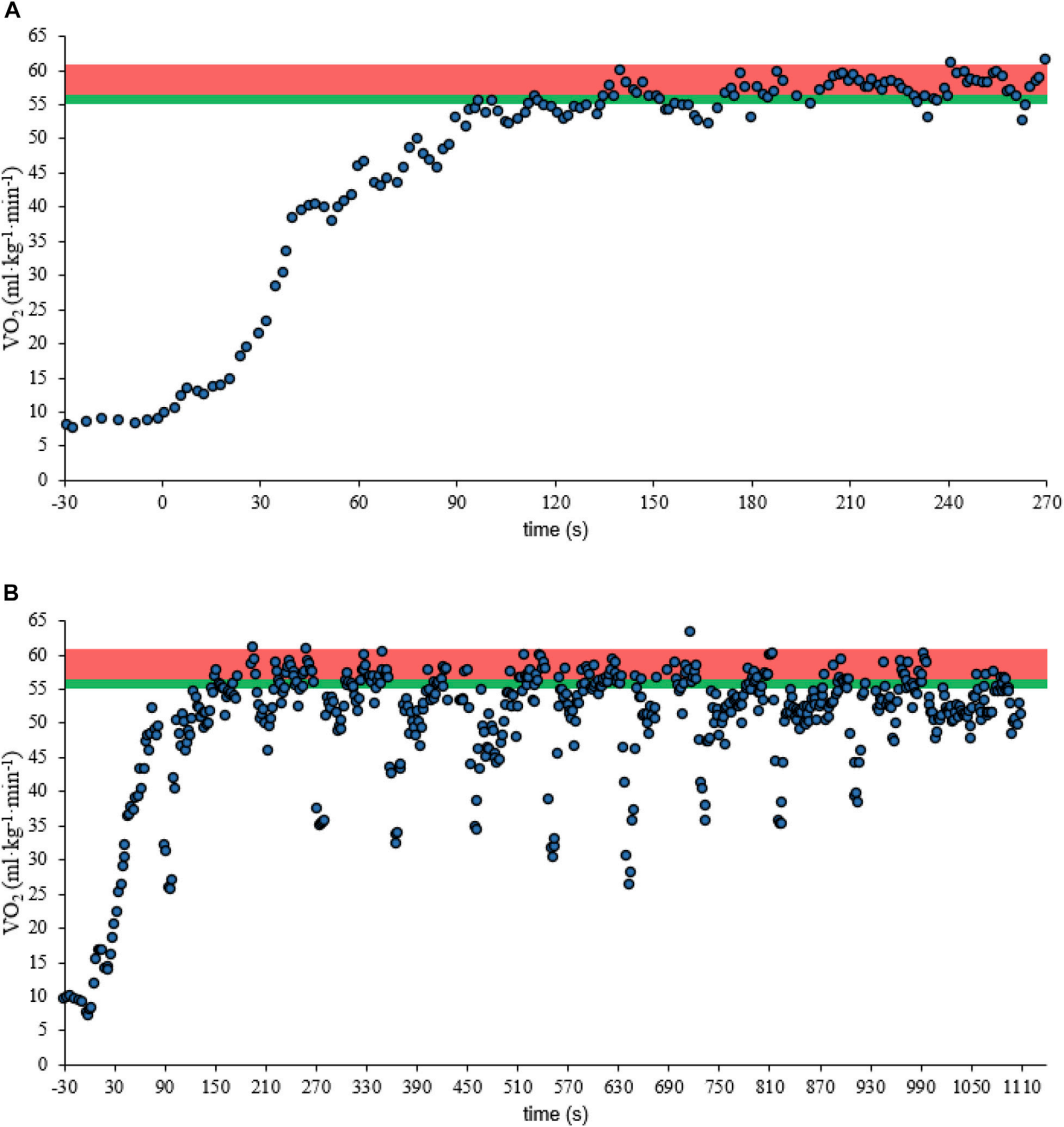
Example of the V̇O_2_ response profiles of the swimmer no 12 in the CT **(A)** and IT_100_ **(B)**. Green and red shadow areas highlight the swimmer t@VT₂ and t@90 %V̇O_2peak_.

The physiological responses during CT and IT_100_ are presented in Table 3 and a typical response of V̇O_2_ is demonstrated in Figure 2.

**TABLE 3:** Mean ± SD of the physiological and performance responses during training trials. N = 22 (9 F, 13 M).

Variable	Training trial		Power	
	Continuous	Intermittent	ρ	Hedges’ g
Peak-V̇O_2_ (ml·kg^−1^·min^−1^)	55.4 ± 5.1	53.1 ± 5.3	<0.149	0.44 [small]
%Peak-V̇O_2_ (%V̇O_2peak_)	100.7 ± 3.1	96.4 ± 4.2*	0.001	1.14 [large]
MPeak-V̇O_2_ (ml·kg^−1^·min^−1^)	—	50.6 ± 4.9		
%MPeak-V̇O_2_ (%V̇O_2peak_)	—	91.8 ± 4.2		
Peak HR (b·min^−1^)	183.2 ± 7.4	182.2 ± 10.4	0.725	0.11 [trivial]
Peak [La^−^] (mmol·l^−1^)	7.8 ± 2.7	7.8 ± 2.8	0.839	0.00 [trivial]
RPE (0–10 units)	8.9 ± 0.9	9.4 ± 0.8	0.051	0.58 [medium]
Distance (m)	315.9 ± 63.3	1,277.3 ± 638.1*	<0.001	2.08 [very large]
t_Lim_ (s)	256.2 ± 60.3	1,013.6 ± 496.6*	<0.001	2.10 [very large]
t@VT₂ (s)	144.1 ± 78.8	448.1 ± 211.1*	<0.001	1.87 [very large]
%t@VT₂ (%)	53.4 ± 20.1	45.8 ± 17.3	0.194	0.40 [small]
t@90%V̇O_2peak_ (s)	127.5 ± 77.1	321.9 ± 208.7*	<0.001	1.21 [large]
%t@90%V̇O_2peak_ (%)	47.3 ± 19.6	34.1 ± 20.9*	0.040	0.64 [medium]
A_p_ (ml·kg^−1^·min^−1^)	42.7 ± 5.3	42.2 ± 3.8	0.758	0.11 [trivial]
TD_p_(s)	12.6 ± 2.2	11.8 ± 2.3	0.297	0.35 [small]
*τ*_p_ (s)	26.5 ± 7.5	25.9 ± 9.4	0.838	0.07 [trivial]
O_2InicialDef_ (ml)	1,658.5 ± 372.2	1,652.0 ± 601.2	0.967	0.01 [trivial]
A_SC_ (ml·min^−1^)	266.2 ± 178.4			
A_SC_ (ml·kg^−1^·min^−1^)	4.0 ± 2.6			
TD_SC_(s)	132.5 ± 20.5			
*τ*_sc_ (s)	39.6 ± 26.4			

Peak-V̇O_2_ and %Peak-V̇O_2_, maximal V̇O_2_ in the test and corresponding percentage to V̇O_2peak_; MPeak-V̇O_2_ and %MPeak-V̇O_2_, average value of the maximal V̇O_2_ achieved in each repetition of the set and corresponding percentage to V̇O_2peak_; Peak [La^−^] and Peak HR, maximal blood lactate concentration and HR, respectively; RPE, rate of perceived exertion; Distance and t_Lim_, maximal distance and time performed by the swimmers; t@VT₂ and t@90%V̇O_2peak_, time spent by the swimmers with V̇O_2_ values above the VT₂ and 90% of the V̇O_2peak_, and corresponding percentage values for the total duration of each test, respectively; A, TD and τ, amplitude, time delay and time constant parameters of the V̇O_2_K, for the primary (p) and slow component phase (Asc); *, statistical differences for the continuous test (*p* < 0.05). The observed sample power for the differences between CT and IT_100_ with regards to %Peak-V̇O_2_, Distance, t_Lim_, t@VT_2_, and t@90% V̇O_2peak_ are 96, 100, 100, 100, and 98%, respectively. For the other variables, The differences between CT and IT_100_ did not attain statistical significance, nor sufficient sample power (i.e., <80%).

The CT presented no significant Peak-V̇O_2_ than the IT_100_ test, but the %Peak-V̇O_2_ is higher in CT. However, the IT_100_ test presented significantly higher values for t_Lim_, distance, t@VT₂, and t@90%V̇O_2peak_. Regarding the time spent near V̇O_2peak_, when the percentage values for the total duration of the sessions were considered, no differences were observed between tests. Also, none of these variables seem to be related between tests.

No differences were found in the V̇O_2_K parameters and O_2InitialDef_ between the CT and the first bout of the IT_100_ test, nor for the peak [La^−^]. However, RPE response is lower for CT than IT_100_. However, both the time constants and the O_2InitialDef_ were correlated between tests (*r* = 0.77 and *r* = 0.67, *p* < 0.01, respectively) and the time constants seem to be highly correlated with the corresponding O_2InitialDef_ (*r* = 0.82 and *r* = 0.92, *p* < 0.01) for CT and IT_100_, respectively.

Both time-limit tests achieve high values of Peak-V̇O_2_, however, only CT reached the V̇O_2peak_ of the incremental test. Moreover, V̇O_2peak_ was highly correlated with Peak-V̇O_2_’s (*r* = 0.95 and *r* = 0.91, *p* < 0.01, for CT and IT_100_, respectively), and Peak-V̇O_2peak_ were also correlated between CT and IT_100_ tests (*r* = 0.88, *p* < 0.01).

The t_Lim_ in the CT presented 1) direct relations with: %t@VT₂ (*r* = 0.56, *p* < 0.01) and %t@90%V̇O_2peak_ (*r* = 0.55, *p* < 0.01); and 2) inverse relations with: MAV (*r* = −0.69, *p* < 0.01) and vVT₂ (*r* = −0.53, *p* < 0.05), which also correlate with each other (*r* = −0.87, *p* < 0.01).

## Discussion

The current study contributed to the literature with the evidence that, as shown previously in running (Demarie et al., 2000), also in swimming the IT_100_ allows the athletes to perform for longer the MAV intensity with longer times spent near the V̇O_2peak_ when compared to the CT, without demanding different blood lactate accumulation and perceived rate exertion. The main evidence of the present study are: 1) both time-limit tests promote high values of V̇O_2_ with considerable times, similar to previous literature findings (Demarie et al., 2000; Almeida et al., 2021), spent near V̇O_2_ maximal values (i.e., ∼53 % and ∼46% of t@VT₂, for CT and IT_100_, respectively), evidencing the training sets efficacy for aerobic improvement, and therefore confirming our first hypothesis; 2) IT_100_ presented a significantly higher t_Lim_ (∼757 s higher), contributing to a significantly higher amount of time spent at or above VT₂ and 90% of V̇O_2peak_ (∼304 and ∼194 s higher, respectively) confirming our second hypothesis; 3) our third hypothesis was not confirmed since faster V̇O_2_ kinetics were not associated with higher t_Lim_, however both time constants were highly associated with the O_2_ initial deficits, suggesting that swimmers with faster kinetics could reduce the anaerobic contribution at the beginning of the exercise.

Demarie et al. (2000), comparing the V̇O_2_ of intermittent and continuous running at 92.2% of MAV, concluded that both have efficacy for endurance training performance, however the authors demonstrated that subjects were truly able to run for a significantly longer time during the intermittent test (∼555 s more), with a significantly longer time with V̇O_2_ values near maximal values (∼316 s more), suggesting that the intermittent test is the best to stimulate the aerobic metabolism at its maximum value. The current results corroborate the reports from Demarie et al. (2000) for running, suggesting that interval training in swimming is more beneficial for developing aerobic power than continuous training. Even though the percentage of V̇O_2peak_ was higher in the continuous test and the percentage of the time performed near maximal V̇O_2_ values was similar between the two training modes in study, the swimmers were able to perform the requested intensity for a significantly longer time in the interval training, which consequently contributed to significantly higher times spent near their V̇O_2_ maximal values. This evidence suggests interval training as the best for stimulating the oxidative system, promoting better chronic adjustments to the aerobic conditioning level of swimmers (Demarie et al., 2000; Bentley et al., 2005; Libicz et al., 2005; Helgerud et al., 2007).

Previous studies reported inverse correlations between the tLim-MAV with MAV and vVT₂ for several exercise modalities (Billat et al., 1996; Billat and Koralsztein, 1996; Faina et al., 1997; Fernandes et al., 2008; Fernandes and Vilas-Boas, 2012). This fact suggests that swimmers with higher aerobic power could not perform an exercise at this intensity for such long times, when compared to swimmers with lower conditioning levels, probably because higher velocities imply a more strenuous effort, leading to fatigue in an earlier stage by the higher anaerobic energy requirements, as suggested by Fernandes et al. (2008). According to Fernandes et al. (2003), this could be explained by distinct phenotypes, which probably influenced the motor unit’s recruitment patterns during the conducted tests, suggesting that swimmers with higher values of second lactate threshold and MAV should use less extensive training sets for aerobic power improvement purposes. Also, Fernandes and Vilas-Boas (2012) reported that the t_Lim_-MAV is influenced by stroking parameters, having a direct relationship with stroke index and stroke length and an inverse correlation with stroke rate. Even though the kinematic parameters were not monitored in this study, it is logical to believe that the same should occur since these variables will influence the swimming economy and contribute to fatigue delay in an earlier test stage. The current study corroborates the inverse relationship between t_Lim_-MAV with MAV and vVT₂, suggesting that high-level swimmers should train with short-distance IT trials at MAV to avoid premature performance deterioration with fatigue in the first trials.

The V̇O_2_ slow component is another factor that can influence the t_Lim_-MAV, however its impact is still an open issue since the literature has been giving contradicting results regarding the relation with the time to exhaustion. Demarie et al. (2001) were the first group to highlight that, as well as in running or cycling, swimming athletes also present V̇O_2_ additional adjustments, as reported in more recent studies (Pessôa-Filho et al., 2012; Reis et al., 2012; Espada et al., 2015) probably because of the effect of fatigue induced by the exercise on the increase in muscle temperature, on muscular contraction characteristics, higher recruitment of motor units (particularly “fast-twitch” fibers), lower mechanical efficiency (associated with the changes on stroking technique), and the energy cost of breathing (which has a higher relevance in swimming) (Fernandes et al., 2003; Espada et al., 2015). Despite the relationships between higher V̇O_2_ slow component with t_Lim_-MAV were not often reported, as in swimming (Demarie et al., 2001) and other modalities (Billat and Koralsztein, 1996; Billat et al., 1998), or in the current study, there are reports showing a direct relationship, suggesting that longer times to exhaustion lead to higher V̇O_2_ slow components (Fernandes et al., 2003; Fernandes et al., 2008; Fernandes and Vilas-Boas, 2012). Such results and the inverse relationship between t_Lim_-MAV and MAV emphasized that the lower maximal aerobic metabolic rate level of swimmers might be related to a larger tolerance at this intensity. Furthermore, this hypothesis suggests that the inverse relationship might be explained by the reliance on anaerobic release, as this is also pointed out by Billat and Koralsztein (1996) and Faina et al. (1997).

Based on the current results, the V̇O_2_K did no influence MAV tolerance nor on the time spent near V̇O_2peak_ during both the continuous and intermittent training modes. This result was unexpected since fast V̇O_2_K response should, theoretically, contribute to the exercise tolerance. However, the correlation found between time constants during continuous and intermittent training modes reinforces the idea that the rate of V̇O_2_ adjustments per se did not influence the tolerance at this intensity, since neither in the continuous nor in the intermittent exercise, no relations with the t_Lim_ were observed. In swimming, several studies also presented no correlation between these two variables for a t_Lim_-MAV test (Fernandes et al., 2003, 2008; Sousa et al., 2014). Moreover, Bailey et al. (2009), testing the effect of an all-out sprint interval training program, concluded that even though both the tolerance to exercise and the V̇O_2_K presented improvements after the program, those two variables were not correlated. In swimming, Almeida et al. (2021) and Bentley et al. (2005) also tested the relation between the time spent near V̇O_2peak_ during intermittent exercise with V̇O_2_K rate of adjustment with no relations found, in agreement with the current findings.

The relation between V̇O_2peak_ and t_Lim_ is also inconsistent in the literature since reports support a direct relationship (Billat and Koralsztein, 1996) and no relationship at all (Demarie et al., 2000; Fernandes and Vilas-Boas, 2012). The lack of a significant correlation shown in the current study is consistent with the assumption that V̇O_2peak_ is directly related to MAV, which is inversely related to the t_Lim_ (Billat et al., 1996; Billat and Koralsztein, 1996; Faina et al., 1997; Fernandes et al., 2008; Fernandes and Vilas-Boas, 2012), as observed in this study.

With regard to the use of the new-Aquatrainer^®^ for the sampling of gas exchange response, it could not be recognized as a limitation for physiological analysis, even when considering that this system delays the actual swimming velocity through the modification of swimming tasks such as turning and gliding (Ribeiro et al., 2016), and supposedly allows a higher contribution of oxidative energetic system than expected during high-intensity short-and middle-trials performances (Campos et al., 2017). Indeed, there are reports stating that a swimmer is able to stroke at a maximum rate when required while wearing new-Aquatrainer^®^, and therefore no impairments are expected for the level of exertion during swimming tests (Ribeiro et al., 2016) and energetic contribution (Almeida et al., 2020; Massini et al., 2021).

## Conclusion

In conclusion, our results suggest: 1) the intermittent training set of 100 m repetitions, with 15 s of rest, is the best training set in order to promote the longest times spent near V̇O_2_ maximal values, and therefore promote gains in V̇O_2peak_; 2) testing the tolerance of swimmers at MAV provides an individualized reference of training intensity, which might assist coaches to manage training for the entire team in conformity with the findings of the current study that higher level swimmers could not perform the MAV intensity longer than swimmers with lower conditioning levels; and 3) that V̇O_2_K seemed not to influence the tolerance at MAV or times spent near V̇O_2peak_ during the continuous and intermittent training modes.

From the current findings, some practical applications are:

• Continuous and intermittent exercises mode at MAV are both able to elicit maximal V̇O_2_ response before exhaustion, and therefore both might be considered suitable training conditions to improve maximal aerobic power.
• The IT_100_ planned at MAV increases considerably the time-limit and time spent near V̇O_2peak_ when compared to continuous longer distances, and therefore considered an advisable exercise mode to preclude earlier exhaustion during such high intensity training.
• The t_Lim_ at MAV might be considered a suitable index of the enhancement of swimming tolerance, and therefore able to parametrize either training efficacy or planning adjustments to engender the physiological chronic alterations required to perform successfully at high aerobic intensities.

When planning training at MAV to improve maximal aerobic power, coaches should consider that the time sustained during CT (∼256 s, in the current study) can be enhanced with IT (∼1,014 s performing ∼12 to 13 bouts of 100 m with a 15 s interval), therefore engendering a longer swimming time with oxidative rates close to maximal values. Following other studies (Billat, 2001; Zuniga et al., 2011; Buchheit and Larson, 2013; Wen et al., 2019; Almeida et al., 2021) this is an effective condition for improving V̇O_2peak._

## Data Availability

The raw data supporting the conclusion of this article will be made available by the authors, without undue reservation.
